# EMG-Based Continuous and Simultaneous Estimation of Arm Kinematics in Able-Bodied Individuals and Stroke Survivors

**DOI:** 10.3389/fnins.2017.00480

**Published:** 2017-08-25

**Authors:** Jie Liu, Sang Hoon Kang, Dali Xu, Yupeng Ren, Song Joo Lee, Li-Qun Zhang

**Affiliations:** ^1^Sensory Motor Performance Program, Rehabilitation Institute of Chicago Chicago, IL, United States; ^2^School of Mechanical, Aerospace, and Nuclear Engineering, Ulsan National Institute of Science and Technology Ulsan, South Korea; ^3^Center for Bionics, Biomedical Research Institute, Korea Institute of Science and Technology, University of Science and Technology Seoul, South Korea; ^4^Department of Physical Therapy and Rehabilitation Science and Department of Orthopaedics, University of Maryland Baltimore, MD, United States; ^5^Department of Bioengineering, University of Maryland College Park, MD, United States

**Keywords:** electromyogram (EMG), non-linear autoregressive exogenous model, continuous decoding, exoskeleton robot, computational neuroscience

## Abstract

Among the potential biological signals for human-machine interactions (brain, nerve, and muscle signals), electromyography (EMG) widely used in clinical setting can be obtained non-invasively as motor commands to control movements. The aim of this study was to develop a model for continuous and simultaneous decoding of multi-joint dynamic arm movements based on multi-channel surface EMG signals crossing the joints, leading to application of myoelectrically controlled exoskeleton robots for upper-limb rehabilitation. Twenty subjects were recruited for this study including 10 stroke subjects and 10 able-bodied subjects. The subjects performed free arm reaching movements in the horizontal plane with an exoskeleton robot. The shoulder, elbow and wrist movements and surface EMG signals from six muscles crossing the three joints were recorded. A non-linear autoregressive exogenous (NARX) model was developed to continuously decode the shoulder, elbow and wrist movements based solely on the EMG signals. The shoulder, elbow and wrist movements were decoded accurately based only on the EMG inputs in all the subjects, with the variance accounted for (VAF) > 98% for all three joints. The proposed approach is capable of simultaneously and continuously decoding multi-joint movements of the human arm by taking into account the non-linear mappings between the muscle EMGs and joint movements, which may provide less effortful control of robotic exoskeletons for rehabilitation training of individuals with neurological disorders and arm impairment.

## Introduction

Rehabilitation robots have been developing rapidly and used for therapeutic training of patients with neurological disorders, including stroke, cerebral palsy, and spinal cord injury (Dipietro et al., 2005; Krebs et al., 2008; Song et al., 2008; Marchal-Crespo and Reinkensmeyer, 2009; Pons, 2010; Frisoli et al., 2012; Heo et al., 2012; Reinkensmeyer and Boninger, 2012; Zariffa et al., 2012; Ren et al., 2013). Advances have been made to build more practical and functional upper-limb powered robotic exoskeleton devices (Nef et al., 2007; Perry et al., 2007; Gupta et al., 2008; Kim et al., 2012; Mao and Agrawal, 2012; Ren et al., 2013; Shao et al., 2014). The advances in powered exoskeletons imply great promise to allow neurologically impaired patients to perform versatile activities, therefore helping restore strength and flexibility of their limbs. In contrast, relatively less attention has been given to providing less effortful control of exoskeleton robots. Previous studies have shown that motor commands are generated by the combination of a small number of muscle synergies, which allows the coordinated recruitment of groups of muscles with specific amplitude balances (Jiang et al., 2010; Overduin et al., 2015; d'Avella, 2016). Muscle synergies can be used to predict the movement of multiple degrees of freedom (DOFs; Ison and Artemiadis, 2014; Jiang et al., 2014a). Controlling a multiple DOFs robotic device requires sophisticated techniques for identification of various movements from recorded electromyography (EMG) signals (Fleischer and Hommel, 2008; Lo et al., 2010; Peerdeman et al., 2011; Scheme and Englehart, 2011; Fougner et al., 2012). A neural control interface is crucial to providing accurate, natural and less effortful control of powered exoskeletons (Kiguchi and Hayashi, 2012; Lenzi et al., 2012).

Among the potential biological signals for human-machine interaction (brain, nerve, and muscle signals), EMG, the ensemble electrical activity of a muscle may be the only experimentally non-invasive record of the motor commands to the muscles that enables routine clinical applications. EMG is generated by the neural activation from the brain and spinal cord and therefore contains substantial movement-related information. It is worth noting that EMG signals do not necessarily reflect the overall computations carried on by the motor system. In fact, they are unlikely to catch neural signals related to a key executive function for shaping motor behavior, i.e., the ability of cancelling pending movements (Mirabella, 2014; Mirabella and Lebedev, 2017). However, in practice EMG signals could enable efficient control of robotic exoskeletons by extracting those motor commands that reach the muscles. Inhibitory control is fundamental for achieving a proper behavioral flexibility due to the fact events cannot be fully predicted practically. In many instances, preplanned actions must be aborted to avoid catastrophic consequences. Often suppression of a planned action occurs within the central nervous system, and thus the related neural activity does not reach the muscles. It is not by chance that brain–machine interfaces enacting inhibitory control have proposed to reproduce goal-directed behaviors in a more naturalistic way recently (Mirabella, 2012; Mirabella and Lebedev, 2017).

Use of EMG in decoding motor commands is one of the most robust and accurate interfaces for controlling robotic devices (Farina and Aszmann, 2014). As a non-invasive measurement containing rich motor control information, surface EMG is an important input for the control of powered robotic devices (Parker et al., 2006; Pons, 2010). As a result, surface EMG is increasingly recognized as one of the important control signals for assistive or rehabilitative devices in robot-aided therapy (Song et al., 2008; Hincapie and Kirsch, 2009; Marchal-Crespo and Reinkensmeyer, 2009; Jiang et al., 2010; Smith and Brown, 2011). Myoelectric control is a promising approach for controlling the multiple DOFs of multifunctional dexterous exoskeletons (Fleischer and Hommel, 2008). However, a major challenge in myoelectric control is to provide simultaneous and proportional control signals for robotic devices with multiple DOFs (Jiang et al., 2010; Fougner et al., 2012). To facilitate a less effortful myoelectric control paradigm, myoelectric controllers should provide proportional control of multiple DOFs simultaneously. This has been addressed in a few recent studies (Ameri et al., 2014a; Farmer et al., 2014; Fougner et al., 2014; Hahne et al., 2014; Ngeo et al., 2014). To provide simultaneous, independent and proportional control of multiple DOFs, various linear and non-linear estimators have been used, including artificial neural networks (Koike and Kawato, 1995; Cheron et al., 1996; Au and Kirsch, 2000; Shrirao et al., 2009; Pulliam et al., 2011; Jiang et al., 2012; Zhang et al., 2012; Ameri et al., 2014b; Farmer et al., 2014; Ngeo et al., 2014), regression techniques (Chen et al., 2013; Ameri et al., 2014a; Hahne et al., 2014), and state-space models (Artemiadis and Kyriakopoulos, 2010, 2011; Pan et al., 2014). Recent research has shown that continuous decoding plays increasingly important role in myoelectric control (Ameri et al., 2014a; Farmer et al., 2014; Fougner et al., 2014; Hahne et al., 2014; Ngeo et al., 2014).

Previous studies have shown that artificial neural networks, being a widely used supervised non-linear approach, outperformed linear regression (a supervised linear approach) and non-negative matrix factorization (a linear unsupervised method) in the EMG decoding paradigm (Hahne et al., 2014; Jiang et al., 2014b). It was possible to predict wrist joint angle instead of forces from EMGs with artificial neural networks when the subject was performing free dynamic movements (Jiang et al., 2012). In particular, a non-linear autoregressive exogenous (NARX) model was utilized to continuously map the kinematics of a transtibial prosthesis and EMG activity to estimate the prosthetic ankle angle in transtibial amputees in a recent study (Farmer et al., 2014). However, few investigators have been able to draw on any systematic research into decoding dynamic multi-joint arm movements. Most studies on EMG decoding have only focused on the estimation of kinematics of the leg and fingers. The purpose of this study was to develop a novel multi-input multi-output decoding method based on the NARX neural network model and predict dynamic multi-joint arm movements simultaneously based only on multi-channel EMG inputs, which can potentially be used to achieve user-friendly, less effortful myoelectric control of robot-aided multi-joint movements.

## Methods

### Subjects

Twenty subjects were recruited for this study including 10 stroke subjects (Table 1) and 10 with no neurological disorder (5 males and 5 females, age: 46.1 ± 14.6 year). All subjects gave written informed consent, which was approved by the Institutional Review Board of Northwestern University. A screening examination and clinical assessment to determine the eligibility for each stroke subject were performed by a physical therapist. Inclusion criteria for participation of the study include: (a) age between 21 and 65 years old; (b) experience of stroke, initial onset > 6 months; (c) medically stable with clearance to participate.

**Table 1:** Physical Characteristics of Subjects.

**Subject no.**	**Age**	**Sex**	**Duration**	**Paretic**	**F-M (UE)**	**MSS**
1	56	M	6	R	36	49.02
2	58	M	1	R	38	54.01
3	59	M	9	R	22	23.00
4	59	M	26	L	16	14.67
5	59	M	11	R	46	60.67
6	63	F	2	L	48	66.02
7	54	F	2	R	21	28.66
8	47	F	4	L	22	19.35
9	45	M	10	L	30	43.33
10	55	F	6	R	50	50.69

***Duration**: years since the onset of stroke. **Paretic:** the side of hemiparesis. **F-M (UE)**: the Fugl-Meyer assessment scale of the paretic upper extremity (total score: 66). **MSS**: the motor status scale measures shoulder, elbow (maximum score = 40), wrist, hand, and finger movements (maximum score = 42)*.

### Experimental procedure

**Figure 1:**
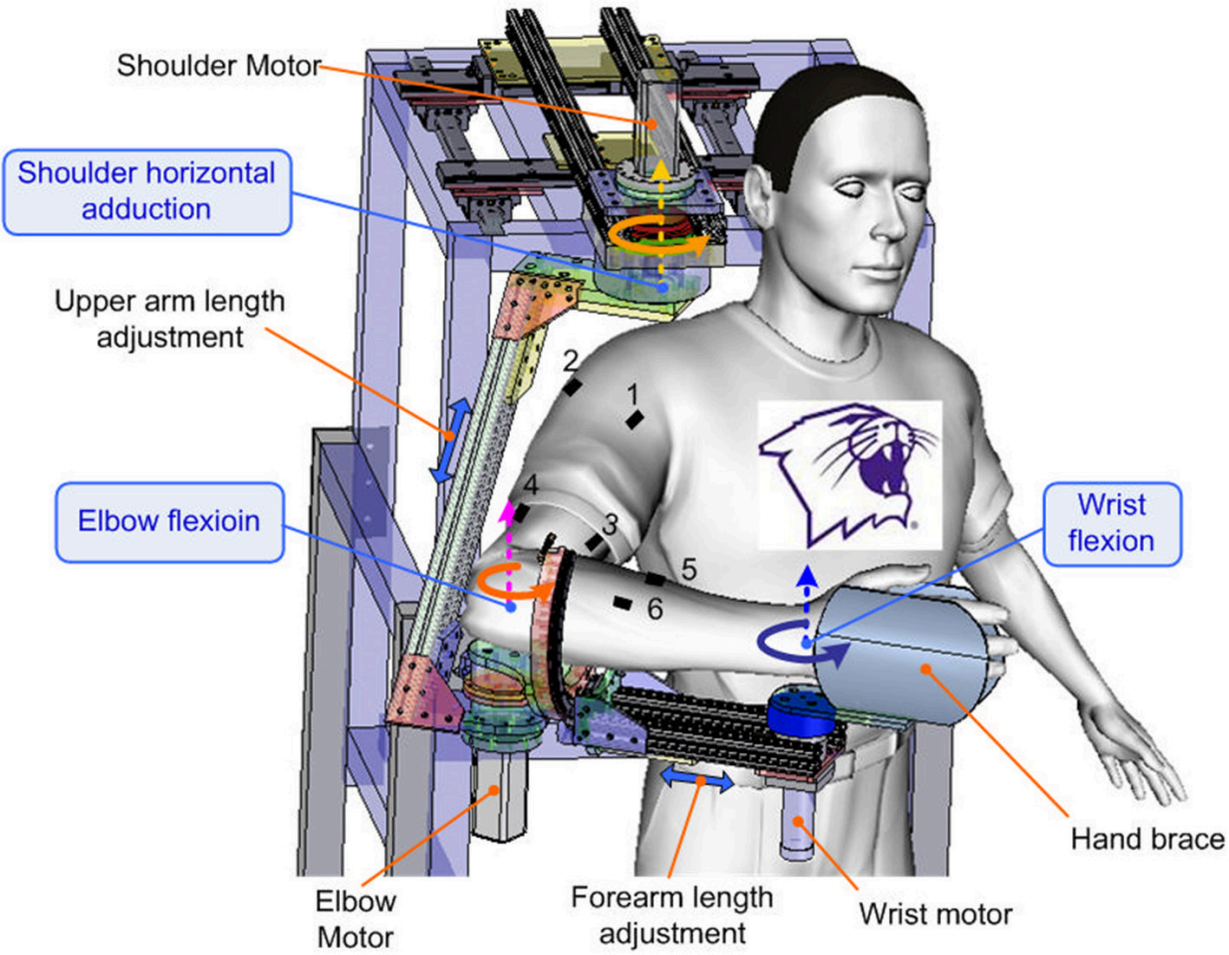
Experimental setup with the subject performing self-paced multi-joint movements with the upper limb exoskeleton robot. The dashed—line arrows mark the axis shoulder horizontal adduction-abduction, elbow flexion-extension, and wrist flexion-extension axes. The double—headed arrows indicate the arm adjustment of the robot. The other degrees of freedom of the robot have been omitted from the figure for clarity. EMG electrodes 1 to 6 were used to record muscle EMGs from the anterior deltoid, posterior deltoid, biceps brachii, triceps long head, flexor carpi radialis, and extensor carpi radialis muscles of the dominant arm of the subject, respectively.

An upper limb exoskeleton robot, called IntelliArm and capable of controlling the shoulder, elbow and wrist individually or simultaneously in the horizontal plane (Ren et al., 2013), was used in the study to conduct supported free arm reaching and movement, as commonly used in stroke rehabilitation (Lo et al., 2010). The subject was seated with the upper arm, forearm, and hand strapped to the IntelliArm through the corresponding braces (Figure 1). The subject's shoulder horizontal adduction-abduction, elbow flexion-extension and wrist flexion-extension axes were aligned with the corresponding mechanical axes of the IntelliArm robot (Ren et al., 2013). The IntelliArm robot was made back-drivable under the internal model based impedance control (IMBIC; Kang et al., 2009), thus the subject could move the arm voluntarily with little resistance from the robot. Six wireless pre-amplified, single differential surface EMG electrodes (Trigno, Delsys Inc; Boston, Massachusetts) were used to record EMG signals from the anterior deltoid, posterior deltoid, biceps brachii, long head of the triceps brachii, flexor carpi radialis, and extensor carpi radialis muscles. The subject was instructed to move their arm voluntarily in the horizontal plane with large concurrent movements across the shoulder, elbow, and wrist joints at the subject's self-selected comfortable speeds (<70 degrees/second), as the speed and movement duration are more likely to vary in real-world settings. The motors used to control the motion of the robot in other planes were locked in this study. The movement range of each of the joints was about 90°. Relative long single trial instead of a few short time trials were used for data recording when movements are freely repeated several times in order to capture both the commonality and the variability of the EMG and motion properties. Thus, the subject performed a single movement trial over a span of about 3 min in this study. Previous studies have used a similar approach for modeling functional relationships between EMG and limb kinematics (Cheron et al., 1996, 2003). The reliability of an experimental study could be affected by several factors such as motivation or boredom. For example, during series of trials, the second one is likely better than the first because subjects intend to improve their performance or because they may benefit from the learning experience of the first one. By contrast, a decreased performance between the first trial and the following trial could possibly be explained by lack of motivation or fatigue (Hopkins, 2000).

### Data collection and signal processing

**Figure 2:**
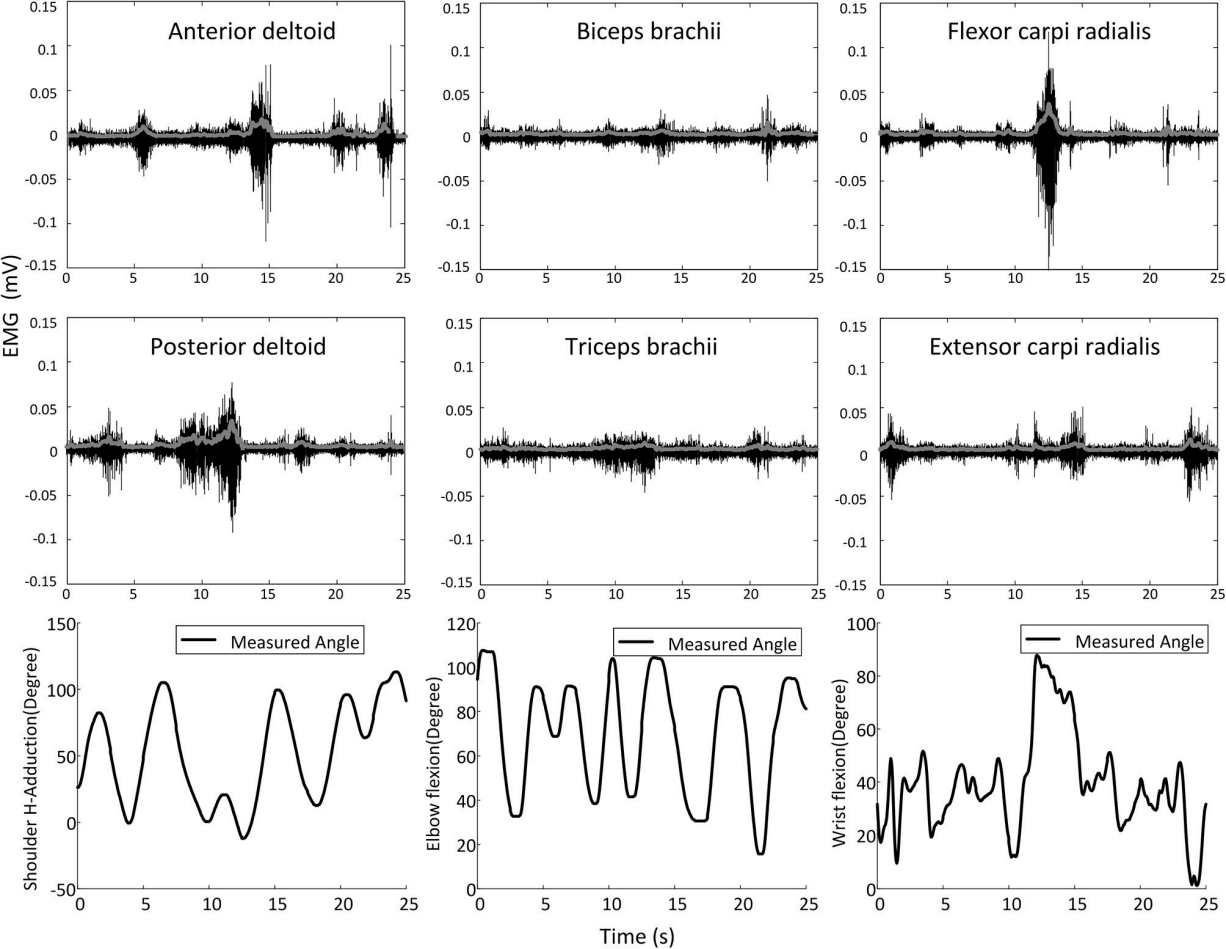
Representative shoulder, elbow, and wrist movements and the corresponding surface EMG signals (raw signals are in black and the corresponding linear envelope profiles are in gray) recorded in a movement trial on one subject.

The surface EMG signals were sampled at 1,000 Hz by using the Trigno EMG system, rectified, and then low-pass filtered with a six-order Butterworth filter (cutoff frequency = 4 Hz) to obtain the linear envelope (LE). Multi-joint position signals from the IntelliArm robot were sampled at 1,000 Hz and synchronized to the EMG data through a trigger signal sent to the aforementioned IntelliArm and EMG data acquisition systems simultaneously. The trigger was implemented as a push button switch. The resultant EMG LE and position signals were then down sampled to 20 Hz for subsequent data analysis. Figure 2 illustrates the representative EMG and kinematic data recorded during a typical experimental trial.

### NARX model

The multi-joint dynamic relationship between the six arm muscles and the shoulder, elbow and wrist movements was characterized by an AutoRegressive with eXogeneous inputs (NARX) model for discrete-time non-linear systems (Leontaritis and Billings, 1985; Billings, 2013) and is represented as

(1) $$\begin{array}{l} y\left(t\right)=f\left[y\left(t-1\right),\ldots ,y\left(t-n_{y}\right),x\left(t-1\right),\ldots ,x\left(t-n_{x}\right)\right]\\ \;+e(t)\left(t\right)\left(t-1\right)t-nx \end{array}$$

where *x(t)* and *y(t)* are the input and output of the model at discrete time step *t*, respectively. While the input order n_x ≥ 1, the output order n_y ≥ 1, and n_x ≤ n_y (Leontaritis and Billings, 1985; Billings, 2013). f(·) is a non-linear function, which can be approximated by a standard multilayer perceptron (MLP) neural network. The resulting connectionist architecture is called a NARX recurrent neural network, which has been used in modeling non-linear dynamic systems (Chen et al., 1990; Lin et al., 1996, 1997; Menezes and Barreto, 2008; Farmer et al., 2014).

Assuming that e(t) has zero mean and finite variance, the minimized mean-square error (MSE) predictor for the NARX model is approximated by

(2) $$\begin{array}{l} \hat{y}\left(t\right)=y\left(t\right)-e\left(t\right)=f[y\left(t-1\right),\ldots ,y\left(t-n_{y}\right),x\left(t-1\right),\\ \;\ldots ,x\left(t-n_{x}\right)] \end{array}$$

The NARX model has a delay structure, creating embedded memory within the neural network. The MSE one-step predictor is a non-linear function of a finite number of past outputs and past inputs.

In contrast to other recurrent networks, NARX neural networks have feedback from the outputs rather than from the hidden states (Siegelmann et al., 1997). As a result, NARX neural networks are able to learn more effectively, converge faster and exhibit better generalizations (Lin et al., 1996, 1997). In addition, the NARX neural networks are computationally as powerful as Turing machines (Siegelmann and Sontag, 1991, 1995), which means that theoretically any problem can be modeled and solved using a NARX neural network. Thus, we used a NARX neural network to predict dynamic arm movements by modeling the non-linear dynamic relationship between the multiple muscle activities and arm kinematics.

### NARX neural network structure

**Figure 3:**
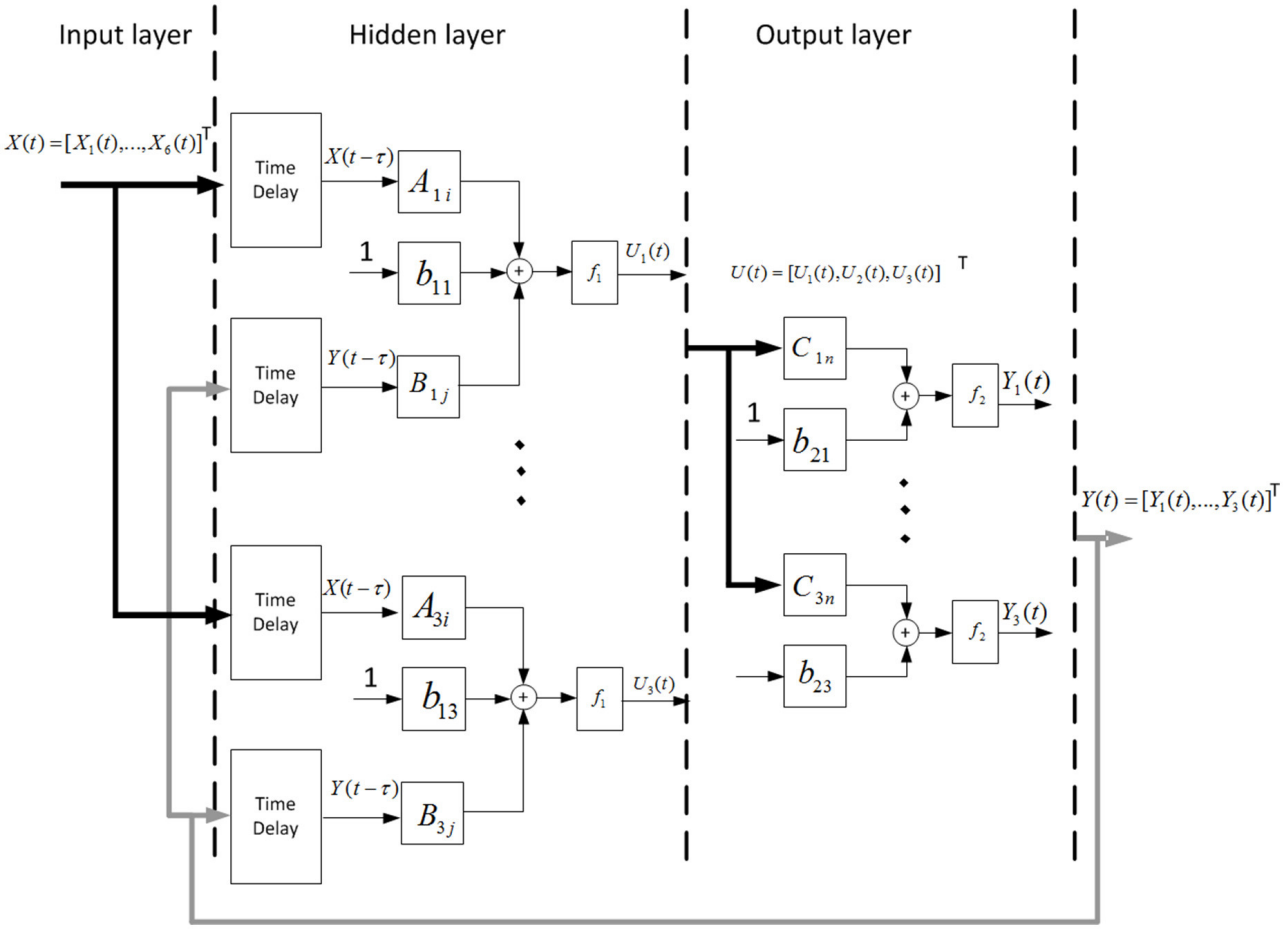
Schematic of the NARX neural network. Measured EMG LE signals and past estimates of the shoulder, elbow, and wrist angles were weighted and fed back via tapped delay lines to the hidden layer consisted of three nodes. Outputs of the hidden layer were weighted and linearly combined to provide continuous estimates of the outputs (shoulder, elbow, and wrist joint angles) over time.

The NARX network model used in this study was developed by using the Neural Network Toolbox in Matlab (Mathworks, Natick, MA), which consisted of an input layer containing the six-channel EMG LE signals recorded from the arm, a hidden layer containing three neurons, and a linear output layer containing three outputs corresponding to the shoulder, elbow, and wrist angular positions (Figure 3).

To continuously decode the multi-joint positions simultaneously from the EMG LE signals, the following NARX network model was used:

(3) $$\begin{array}{l} U_{n}\left(t\right)=f_{1}(\sum_{i=1}^{N_{x}}\sum_{k=0}^{n_{x}}A_{ni}\left(k\right)X_{i}\left(t-m-k\right)\\ \;-\sum_{j\;=1}^{N_{y}}\sum_{k=0}^{n_{y}}B_{\text{nj}}\left(k\right)Y_{j}\left(t-m-k\right)+b_{\text{1n}}),\\ \;n=1,2,3 \end{array}$$

(4) $$Y_{j}\left(t\right)=f_{2}\left(\sum_{n=1}^{3}C_{\text{jn}}\left(t\right)U_{n}\left(t\right)+b_{\text{2j}}\right),\;j=1,2,3$$

where *U*_*n*_(*t*) represents output of one of the non-linear nodes of the hidden layer, *Y*_*j*_(*t*) is the shoulder, elbow or wrist angular position predicted by the NARX network model at time *t*; *N*_*x*_ (= 6) is the number of EMG LE signals; *n*_*x*_ (= 2) is the number of past input time lags used in the NARX model; *N*_*y*_ (= 3) is the number of joint angles, *n*_*y*_ (= 2) is the number of past output time lags used in the NARX model; *m* (= 2) is the number of time lags, which specifies the embedding delay (τ = mΔt) and correspond to the prediction interval; *X*_*i*_(*t* − *m* − *k*) is the ith EMG LE at time lag *m* + *k*; *Y*_*j*_(*t* − *m* − *k*) is a joint angle predicted by the NARX model at time lag *m* + *k*; *A*_*ni*_, *B*_nj_ and *C*_jn_ are the weight vectors. *b*_1n_ and *b*_2j_ are the bias weights. *f*_1_ and *f*_2_ are the sigmoid function and linear function, respectively.

The NARX neural network model has 51 parameters in total, which are estimated in training the neural network offline by using a supervised learning procedure to minimize the errors between the NARX network model outputs and experimentally measured angles.

### Performance measure

For each subject, a five-fold cross-validation procedure was used to evaluate the performance. The EMG and arm joint kinematic data were divided into five segments with equal length. Each time, four out of five segments of data were used as the training set and the remaining segment was used as the validation set (training: 80%, validation: 20%). The procedure was repeated with each of five segments used as the validation set once. To evaluate the performance of the trained decoder, the variance accounted for (VAF) was calculated between the measured and decoded joint angles based on the fresh data (Kearney and Hunter, 1990; Fagg et al., 2009).

One-way ANOVA with repeated measures was utilized to compare the decoding performance across the multiple joints. The group factor had three levels, corresponding to the shoulder, elbow, and wrist joints. The null hypothesis was that there was no difference between the decoding performance of the shoulder, elbow and wrist joints. Multiple comparisons with Bonferroni correction were conducted to compare pairwise differences between the joints.

## Experimental results

### Decoding performance and the effect of prediction interval

**Figure 4:**
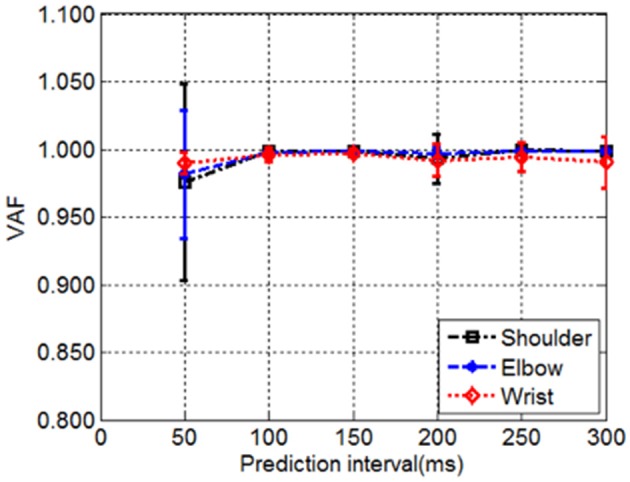
The decoding performance demonstrated by the VAF as a function of prediction interval. The VAF between measured and decoded shoulder, elbow and wrist joint angles over the tested prediction intervals were averaged across all subjects. The vertical bars gave the corresponding standard deviations.

The effect of the length of prediction interval on the decoding performance was evaluated as suggested by previous research (Farmer et al., 2014). The relationship between decoding accuracy and the prediction interval was plotted for the shoulder, elbow, and wrist joint angles predicted based on only six muscle EMG signals, which shows that the decoding performance varies with the length of prediction interval (Figure 4). An increase of window length from 50 to 100 ms led to consistent improvement in decoding performance across the joints, as indicated by the higher VAF. A further increase of the prediction interval to 300 ms was accompanied with a slight decrease in the decoding performance. For each prediction interval, no significant differences were observed between different joints [One-way repeated measures ANOVA, *F*_(2, 15)_ = 0.16, *P* = 0.85, partial eta squared ${\eta_{p}}^{2}$ = 0.02]. Averaged across all the able-bodied subjects, the prediction interval of 100 ms yielded the best performance with the highest average VAF, which was used in the subsequent analysis for decoding.

### Position decoding results

**Figure 5:**
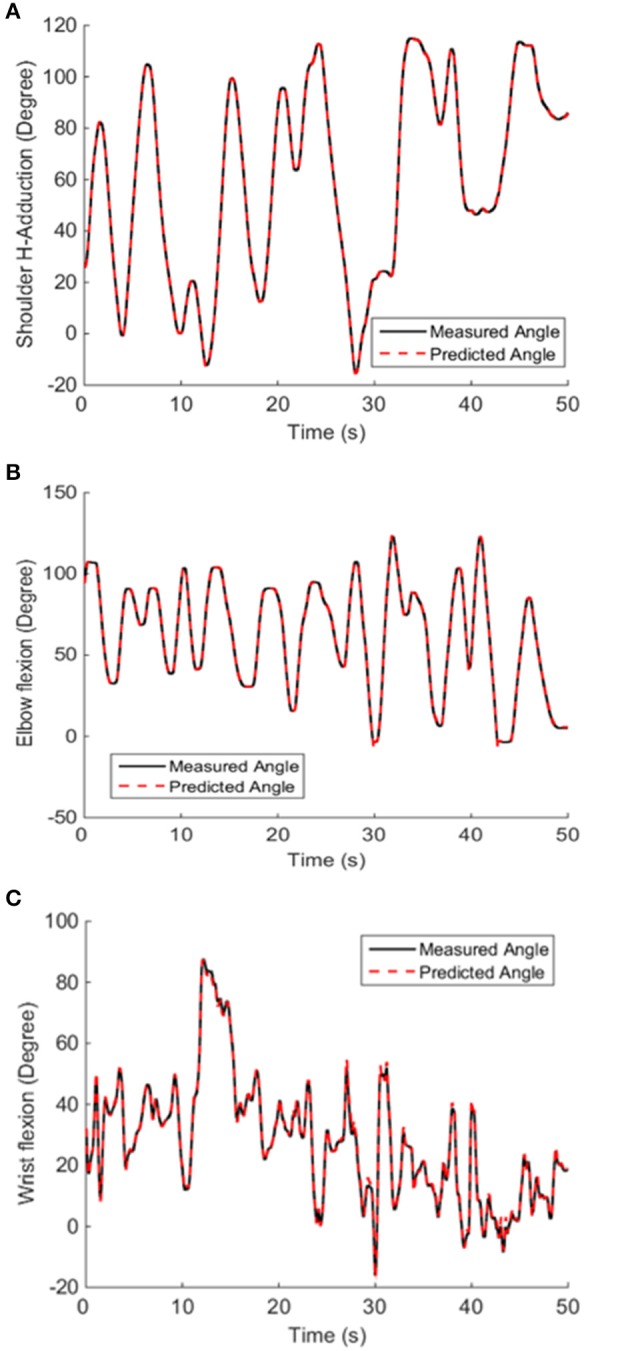
Decoded and measured shoulder **(A)**, elbow **(B)**, and wrist **(C)** joint angles from a representative subject. The NARX neural network accurately predicted actual kinematics with the prediction interval of 100 ms. The VAF of the predicted shoulder, elbow and wrist position calculated from fresh validation data (50 s long) were 0.9999, 0.999, and 0.9977, respectively.

The offline decoding performance of NARX neural network was assessed in terms of its performance to decode the shoulder, elbow and wrist joint positions during active movements using only the six muscle EMG LEs as inputs. High performance in position decoding was achieved for all subjects by using the NARX model, which has a hidden layer containing three neurons, and a linear output layer containing the shoulder, elbow and wrist angle outputs with the prediction interval of 100 ms. Joint angle predictions made using the NARX neural network model based on only the multiple EMG inputs matched very closely with the measured shoulder, elbow, and wrist positions (Figure 5).

**Figure 6:**
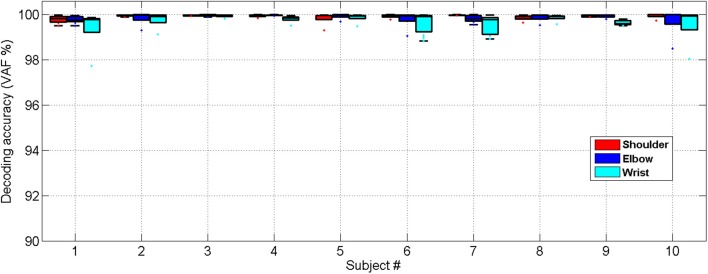
Cross-validated position decoding performance characterized by the VAF over all able-bodied subjects.

Very close matches between the decoded and measured joint angles were achieved for all 10 able-bodied subjects, as shown in Figure 6. The ANOVA showed a significant difference among the decoding performances for the shoulder, elbow and wrist joints [One-way ANOVA, *F*_(2, 18)_ = 5.61, *P* = 0.0092, partial eta squared ${\eta_{p}}^{2}=$ 0.29]. The decoding accuracy for the wrist was lower than that achieved for the shoulder and elbow (Bonferroni test, *P* < 0.05).

**Figure 7:**
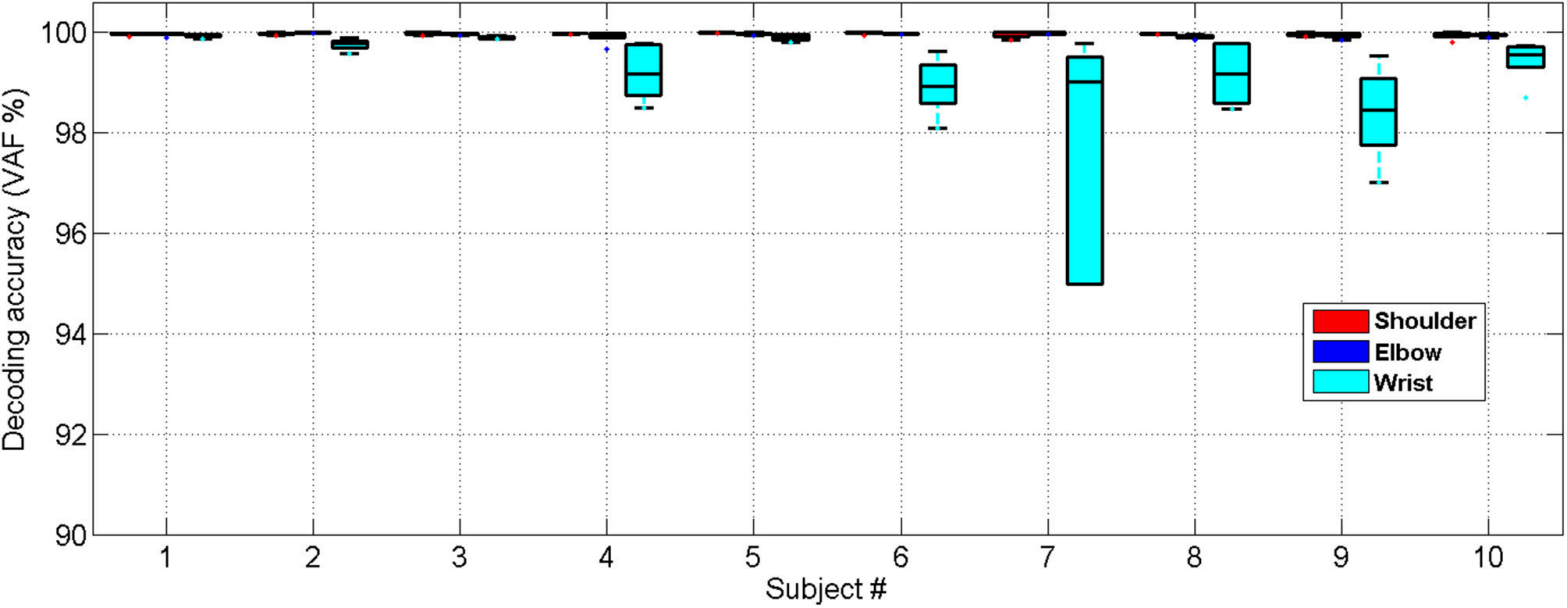
Cross-validated position decoding performance characterized by the VAF over all subjects post stroke.

Similarly, we have further tested the feasibility of the NARX decoder in 10 stroke subjects showing that the decoded and measured joint angles were close for all 10 stroke subjects who had various levels of functional impairment (Motor status scale 14.67–66.02, Fugl-Meyer score 16–50), as shown in Figure 7. The ANOVA showed a significant difference among the decoding performances for the shoulder, elbow and wrist joints [One-way ANOVA, *F*_(2, 18)_ = 7.31, *P* = 0.0029, partial eta squared ${\eta_{p}}^{2}=$ 0.35]. The decoding accuracy for the wrist was lower than that achieved for the shoulder and elbow (Bonferroni test, *P* < 0.05).

## Discussion and conclusion

The study investigated decoding multi-joint dynamic arm movements based solely on surface EMG signals from muscles crossing the joints by extracting those motor commands that reach the muscles. This study contributes to rapidly growing research on myoelectric control of limb movements by demonstrating continuous decoding of arm kinematics using surface EMG signals based on the NARX model. In the current study, taking into account the non-linear dynamical relationship between the muscle EMGs and arm movements, we successfully estimated shoulder, elbow and wrist joint movements using only the six EMG signals by adopting time delays and feedback stream into an artificial neural network. These results are consistent with recent studies (Jiang et al., 2012; Farmer et al., 2014; Hahne et al., 2014; Ngeo et al., 2014) in which proportional and simultaneous estimation of limb kinematics was achieved toward myoelectric control, and suggest the use of EMG as a robust and accurate interface for robot control (Farina and Aszmann, 2014). These findings further support the approach to decoding dynamic limb movements using surface EMG signals by taking advantage of artificial neural networks.

Motivated by the fact that NARX neural networks have a recurrent dynamic nature and are computationally equivalent to Turing machines (Siegelmann and Sontag, 1991, 1995), we assessed the NARX networks for continuous decoding of multi-joint dynamic arm movements using surface EMG signals. The performance of the decoder was evaluated by offline analysis using EMG signals from the six muscles and kinematic data measured by the robot during dynamic multi-joint arm movements in the horizontal plane. The decoding accuracies in terms of VAF, were above 98% for the shoulder, elbow and the wrist joints possibly due to the fact that the noise of EMG recordings was suppressed by low-pass filtering the EMG signals with a six-order Butterworth filter (cutoff frequency = 4 Hz). The decoding performance of the wrist was slightly inferior to that obtained for the shoulder and elbow, possibly because the kinematic data collected from the wrist joint were noisy due to the relatively large noise caused by the Harmonic Drive gear compared to the torque generated by subjects during the voluntary wrist movement under impedance control. This finding is in agreement with that reported by Farmer et al. who showed a NARX model could continuously estimate the prosthetic ankle angle in transtibial amputees using the EMGs recorded from the residual lower limb (Farmer et al., 2014). Furthermore, the method is able to continuously decode movements of multiple joint simultaneously instead of a single joint movement.

Previous studies with able-bodied individuals showed promising results, which need to be interpreted with caution, as the findings might not be directly transferable to neurologically impaired patients, because voluntary surface EMG signals might be contaminated by spontaneous motor activity in studies involving neurological impairments. For instance, when recording EMG signals from paretic muscles of stroke or spinal cord injury patients, abnormal hyper-excitable motor unit discharges may induce spontaneous spurious spikes, compromising the voluntary EMG signals (McKay et al., 2011). Previous studies with incomplete spinal cord injury have shown that the resulting involuntary background spikes can contaminate surface EMG recordings thus result in inappropriate interpretation of the signals (Liu et al., 2014a,b). Therefore, we have further tested the feasibility of the NARX decoder in 10 stroke subjects to evaluate the current method on neurologically impaired patients, we observed that the high decoding accuracy (VAF > 98% for all three joints) was consistent across different stroke subjects who had various levels of functional impairment (Motor status scale 14.67–66.02, Fugl-Meyer score 16–50). It was observed that the stroke subjects have more variable decoding results compared to the healthy subjects with respect to the wrist joint angle, In contrast, the healthy subjects have more variable decoding results for the shoulder and elbow joints, This result may be explained by the fact that the healthy subjects have bigger motion ranges, high speeds and velocity changes. These stroke subjects might have different clinical features, because they are very likely of having undergone very different degree of brain plasticity. We observed that nothing would change by removing patients with more than 10 years since the onset of stroke. However, with a small sample size, caution must be applied, as the findings might not be extrapolated to all patients with different clinical features.

Note that unlike the time delay neural network (TDNN) approach for predicting joint-angle trajectories based on EMG signals during arm movements (Au and Kirsch, 2000; Pulliam et al., 2011; Smith and Brown, 2011), the NARX network makes full use of the model output feedback and has powerful modeling, learning and generalization capacities (Chen et al., 1990; Lin et al., 1996, 1997; Menezes and Barreto, 2008; Farmer et al., 2014). The presence of direct feedback from the output gives strong decoding power to the NARX model. More specifically, the NARX model makes use of the AR model's implicit characterization of the limb kinematics vis-à-vis the feedback. In contrast, the absence of such a feedback in the TDNN model makes it less powerful in decoding. Thus, the NARX network outperforms conventional neural network based decoders, such as the TDNN network (Menezes and Barreto, 2008) qualitatively. Despite this, we acknowledge that future systematic comparison studies on different decoding methods are needed.

EMG signals have been used to control joint angular velocity indirectly by commanding the stiffness equilibrium angle (Ha et al., 2011). However, from a practical point of view, models characterizing multiple muscles controlling human joints are rather complex even for a single joint due to the complex nature of neuromusculoskeletal systems, and usually they require exhaustive, time-consuming offline analysis of neuromusculoskeletal system identification problem (Koo and Mak, 2005). Thus, using such models for real-time decoding is difficult and their real-time application remains to be demonstrated, especially for applications to neurologically impaired cases.

Despite these promising results, questions remain. A limitation is that the high accuracy may be based on the fact that the subjects was training in performing a simple and repetitive arm movement and the arm movements were restricted to a horizontal plane with arm-weight-supported, as opposed to 3-D arm movement. Horizontal plane arm movements have been widely studied and commonly used in rehabilitation of patients post stroke (Hollerbach and Flash, 1982; Lo et al., 2010). It could be argued that the positive results were due to the repeated simplified movements in 2-D space. Still, further research should be done to investigate different arm movements in 3-D space without arm-weight-support. Furthermore, although the IntelliArm robot was controlled to be backdrivable, it might still induce some external loading to the subject's arm during the multi-joint arm movements, which might affect the EMG signals and the myoelectric control behavior of human arm movement. Everyday tasks involve behaviors uncertainty originating from a large variety of sources, which in some circumstances might require to suppress preplanned actions (Mirabella, 2014). However, in this study the presence of behaviors uncertainty was very limited if not completely absent. Therefore, it was not possible to examine the EMG signal variability in the presence of unpredictable changes. This is an important issue for future research.

In summary, the proposed approach is capable of continuously decoding multiple joint movements of the human arm simultaneously by taking into account the non-linear mappings between the multi-joint arm kinematics and multiple muscle EMGs, which may help improve myoelectric control of robotic exoskeletons and robot-guided arm rehabilitation in neurological disorders.

## Ethics statement

Twenty subjects were recruited for this study including ten stroke subjects (Table 1) and ten with no neurological disorder (5 males and 5 females, age: 46.1 ± 14.6 year). This study was carried out in accordance with the recommendations of guidelines from the Collaborative Institutional Training Initiative (TICI) program, and the Institutional Review Board of Northwestern University. All subjects gave written informed consent, which was approved by the Institutional Review Board of Northwestern University.

## Author contributions

Conceived and designed the experiments: JL and LZ. Performed the experiments and data acquisitions: JL, SK, SL, DX, and YR. Analyzed and the data interpretation: JL, DX, YR, and LZ. Contributed reagents/materials/analysis tools: JL, SK, DX, YR, and SL. Wrote the paper: JL and LZ. Final approval of the version to be published: JL and LZ.

### Conflict of interest statement

LZ and YR hold equity positions in Rehabtek LLC, which is involved in developing the rehabilitation robot used in this study. The other authors declare that the research was conducted in the absence of any commercial or financial relationships that could be construed as a potential conflict of interest.
